# The glomus tumor resorbed bone and teeth in the mandible: a case report

**DOI:** 10.1186/s13005-018-0175-3

**Published:** 2018-09-25

**Authors:** Kazuto Kurohara, Yasuyuki Michi, Akane Yukimori, Satoshi Yamaguchi

**Affiliations:** 1 Department of Oral and Maxillofacial Surgery, Graduate School, Mie University, 174, Edobashi 2-chome, Tsu-shi, Mie 514-8507 Japan; 2 Department of Maxillofacial Surgery, Division of Maxillofacial and Neck Reconstruction, Graduate School of Tokyo Medical and Dental University, Tokyo, Japan; 3 Department of Oral Pathology, Division of Oral Health Sciences, Graduate school of Tokyo Medical and Dental University, Tokyo, Japan

**Keywords:** Case report, Glomus tumor, Resorption of teeth roots, Recurrence, Mandible

## Abstract

**Background:**

A glomus tumor is a rare neoplasm usually found in the dermis or subcutaneous tissue of the extremities. It is rare for the glomus tumor to occur on the head and face. Only 26 glomus tumors of the oral region and affected bone have been reported in the English-language literature (Table 1). We report a case of a glomus tumor at the mandible. As a new point, the glomus tumor resorbed a bone and teeth roots when the tumor progressed into the mandible.

**Case presentation:**

The patient was a 44-year-old Japanese man who complained swelling of the right mandible. Radiographic examination showed a multilocular radiolucency area in the left mandible. Radiographic findings on our case resembled those of a common benign tumor. The lesion occupied to the premolar and molar area and revealed that the tumor resorbed the roots of the teeth. The lesion was removed surgically with the buccal cortical bone and buccal mucosa in contact with the mass of the tumor. The mass fully excised intraorally under general anesthesia, and the inferior alveolar nerve in contact with the mass was preserved.

The specimen was pathologically diagnosed as a glomus tumor. Immunohistochemical staining was positive for vimentin, muscle-specific actin/HHF35, and calponin. A hairline-shaped area of positive staining for type IV collagen surrounding the tumor cells was also observed. In contrast, staining for alpha-SMA, cytokeratin (AE1/AE3), cytokeratin (CAM5.2), CK19, CD31, CD34, CD68, p63, S-100, Factor VIII, and desmin was all negative. The Ki-67 labeling index was almost 1%.

A recurrent tumor was again detected in the site below the primary tumor at an 8-year follow-up, and it was surgically removed. The patient has had no symptoms of recurrence in 2 years after the second operation.

**Conclusion:**

The glomus tumor resorbed a bone and teeth roots when the tumor progressed into the mandible. The immunohistochemical features of the tumor were consistent with those described in previous reports. It is important to completely remove the Glomus tumor.

## Background

A glomus tumor is a rare neoplasm usually found in the dermis or subcutaneous tissue of the extremities, where it causes localized pain, tenderness, and cold sensitivity [1]. The glomus body is a contractile neuromyoarterial receptor that regulates peripheral blood flow and temperature [2]. Glomus tumors constitute one or more components of the glomus body and are accordingly identified as malformations of the neuromyoarterial system [1]. Glomus tumors with three distinctive cellular appearances have been described [3]. Type I, the mucoid-hyaline type, is characterized by hyalinized connective tissue interspersed with islands of glomus cells. Type II, the type most frequently described as a glomus tumor, consists of solid masses of glomus cells with limited vascular and connective tissue components. Type III, the angiomatous type, is recognized by its abundance of vascular structures. According to the World Health Organization Classification of Tumors, glomus tumor can be divided into 3 subtypes based on the predominance of a cellular component (solid glomus tumor), a smooth muscle component (glomangiomyoma), or a vascular component (glomangioma) [4].

In this article, we present a case of a glomus tumor of the mandible. Glomus tumors localized in the oral cavity have been reported in only 26 cases in the medical literature, and our case is the first glomus tumor localized in the mandible and affected teeth. The purposes of this article are to present details of this rare glomus tumor of the mandible, describe the pathological features revealed by immunohistochemical study, and review the literature on glomus tumors affecting the oral region.

## Case presentation

A 44-year-old Japanese man presented to the Maxillofacial Surgery Clinic at the Tokyo Medical and Dental University, Tokyo, Japan, with reports of a hard mass and dull pain in the left mandible. His medical history was generally unremarkable, though his mandible had been accidentally smashed against his young son’s head at the age of 40. No fracture was detected at the time, and no treatment was received.

**Fig. 1:**
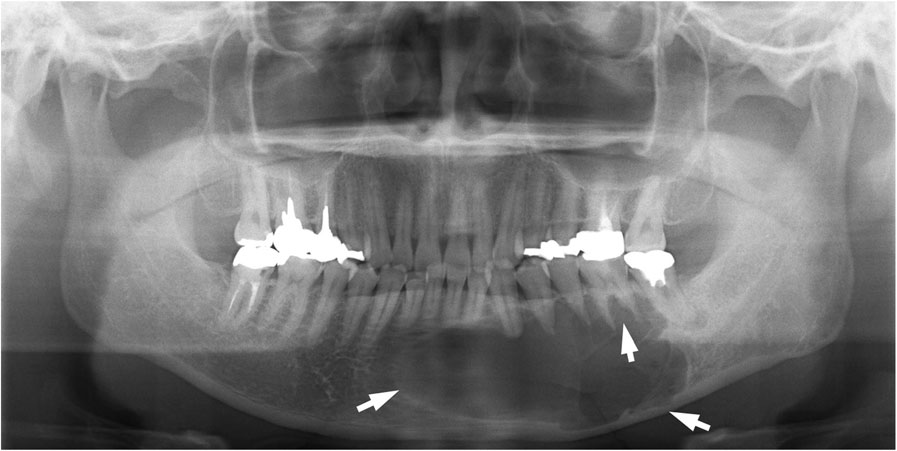
A panoramic radiograph exhibiting a multilocular radiolucency in the region of the mandibular ranged from the front teeth to the first molar (arrows), and the lesion caused the resorption of the adjacent teeth roots

**Fig. 2:**
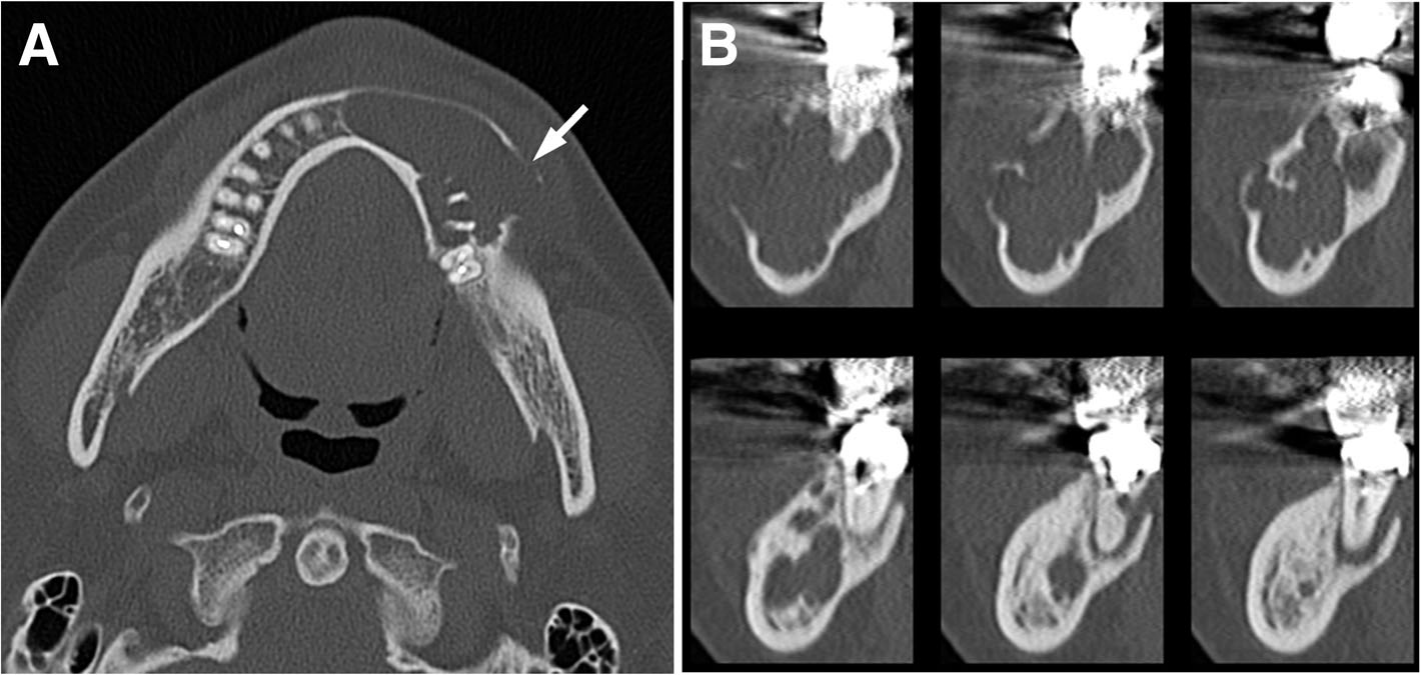
Preoperative CT scan showing mass measuring 45 × 30 × 30 mm. Axial CT bone window image showed that there was a cortical bone thinning or partial resorption around the second premolar of the mandible (arrows). Coronal CT scan showing cortical bone loss and resorption of the first molar roots of the mandible

Clinical examination revealed slight swelling in the left lower molar region and swelling of the left mandible. There was no disturbance of sensitivity in the left lower lip or chin, and he could move his lips normally. Radiographic examination showed large, irregular, multilocular radiolucency of the left area of mandible extending to the premolar and molar area, with no evidence of any impacted tooth (Fig. 1). A computed tomography (CT) image revealed a 45 × 30 × 30 mm multilocular cystic mass in the mandible (Fig. 2A). An axial CT image showed thinning or partly resorption site of the buccal cortical plate. The coronal section images of the CT scan showed the mass lesion displacing the mandibular canal downward, near the inferior border. The roots of the adjacent teeth were resorbed, changing their shapes (Fig. 2B). The location of the teeth was not changed.

**Table 1:** Features of glomus tumor affecting the oral cavity as reported in the literature

Author	year	age	gender	anatomic location	IHC profile
Von Langer [33]	1949	52	M	hard palate	Not available
King [34]	1954	32	M	gingiva	Not available
Kirschner and Strassburg [35]	1962	56	M	gingiva/ alveolar mucosa	Not available
Grande and D’Angelo [36]	1962	42	M	hard palate	Not available
Frankel [37]	1965	13	M	buccal mucosa	Not available
Harris and Griffin [38]	1965	35	F	periodontium/ gingiva	Not available
Sidhu and Subherwal [39]	1967	10	F	hard palate	Not available
Charles [40]	1976	17	F	hard palate	Not available
Sato et al. [41]	1979	29	M	tongue	Not available
Tajima et al. [42]	1981	63	F	tongue	Not available
Saku et al. [43]	1985	45	M	buccal mucosa	actin(+), smooth muscle myosin(+)
Ficarra et al. [44]	1986	51	F	upper lip	Not available
Moody et al. [45]	1986	65	F	upper lip	Vimentin(+), factor VIII(−), CD45(−), A-BgA(−), cytokeratin(−)
Stajcic and Bojic [46]	1987	55	M	tongue	Not available
Tokiwa et al. [47]	1990	36	M	gingiva of mandibular	Not available
Geraghty et al. [19]	1992	71	M	hard palate	alpha actin(−), neuron-specific enolase(−), chromogranin(−), desmin(−)
Kusama et al. [48]	1995	57	M	upper lip	S-100(+), actin(+), desmin(+), vimentin(+), factor VIII(−)
Sakashita et al. [49]	1997	54	M	upper lip	Vimentin(+), smooth muscle actin(+), factor VIII(−)
Yu et al. [11]	2000	54	F	left mandibular area, lip, anterior buccal mucosa	smooth muscle actin(+), S-100(−)
Kessaris et al. [8]	2001	46	F	hard palate	Vimentin(+), smooth muscle actin(+), actin(−), desmin(−), chromogranin(−), neuron-specific enolase(−), epithelial membrane antigen(−), cytokeratin(−), factor VIII(−)
Rallis et al. [7]	2004	85	F	upper lip	smooth muscle actin(+), muscle specific actin(+), vimentin(+), desmin(−), S-100(−), epithelial membrane antigen(−), neuron-specific enolase(−), AE1/3(−), Leu7(−), CD3,CD31,CD34,CD45,CD20(−), cytokeratin(−)
Quesada R et al. [50]	2004	61	M	tongue	Not available
Lanza et al. [51]	2005	65	M	lower lip	Not available
Maeda et al. [52]	2005	20	M	palate	Vimentin(+), smooth muscle actin(+), HHF35(+), keratin(−), S-100(−), factor VIII(−), desmin(−)
Boros et al. [10]	2009	34	M	lower lip	smooth muscle actin(+), muscle specific actin(+), S-100(+), kerarin(−), epithelial membrane antigen(−), CD34(−), CD31(−), chromogranin(−)
Derand III et al. [53]	2010	11	F	lower lip	pan-cytokeratin(−), vimentin(+), smooth muscle actin(+), S-100, factor VIII(−)
current case	2016	44	M	mandible	Vimentin(+), muscle specific actin/HHF35(+), calponin(+), typeIV collagen(+), smooth muscle actin(−), cytokeratin(AE1/AE3)(−), cytokeratin(CAM5.2)(−), CK19(−), CD31(−), CD34(−), CD68(−), p63(−), S-100(−), factor VIII(−), desmin(−)

The initial clinical impression was an ameloblastoma, myxoma, keratocystic odontogenic tumor or another tumor type lesion.

The lesion was removed surgically with the buccal cortical bone and buccal mucosa in contact with the mass of the tumor. The mass fully excised intraorally under general anesthesia, and the inferior alveolar nerve in contact with the mass was preserved.

**Fig. 3:**
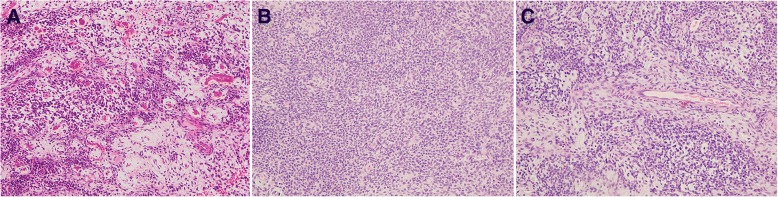
Histologic aspects of the primary glomus tumor. Photomicrograph showing small blood vessels surrounded by round tumor cells with round/oval nuclei and eosinophilic cytoplasm. **a** Tumor with myxoid change, H&E stain, × 200, (**b**) Solid sheets of tumor cells, H&E stain, × 200, (**c**) Tumor cells surrounded small blood vessels, H&E stain, × 200

The resected specimen of the primary tumor was a soft, fragile, yellowish-white mass. Microscopically, tumor tissues were composed of plexiform or cord-like nests of tumor cells with round to oval nuclei and eosinophilic cytoplasm in a matrix with prominent myxoid change (Fig. 3A). On the other hand, the component of solid sheets of tumor cells was limited (Fig. 3B). In some areas, tumor cells surrounded small blood vessels (Fig. 3C). Moreover, tumor cells manifested a uniform cell morphology, poor dysplasia, and inconspicuous mitosis.

**Fig. 4:**
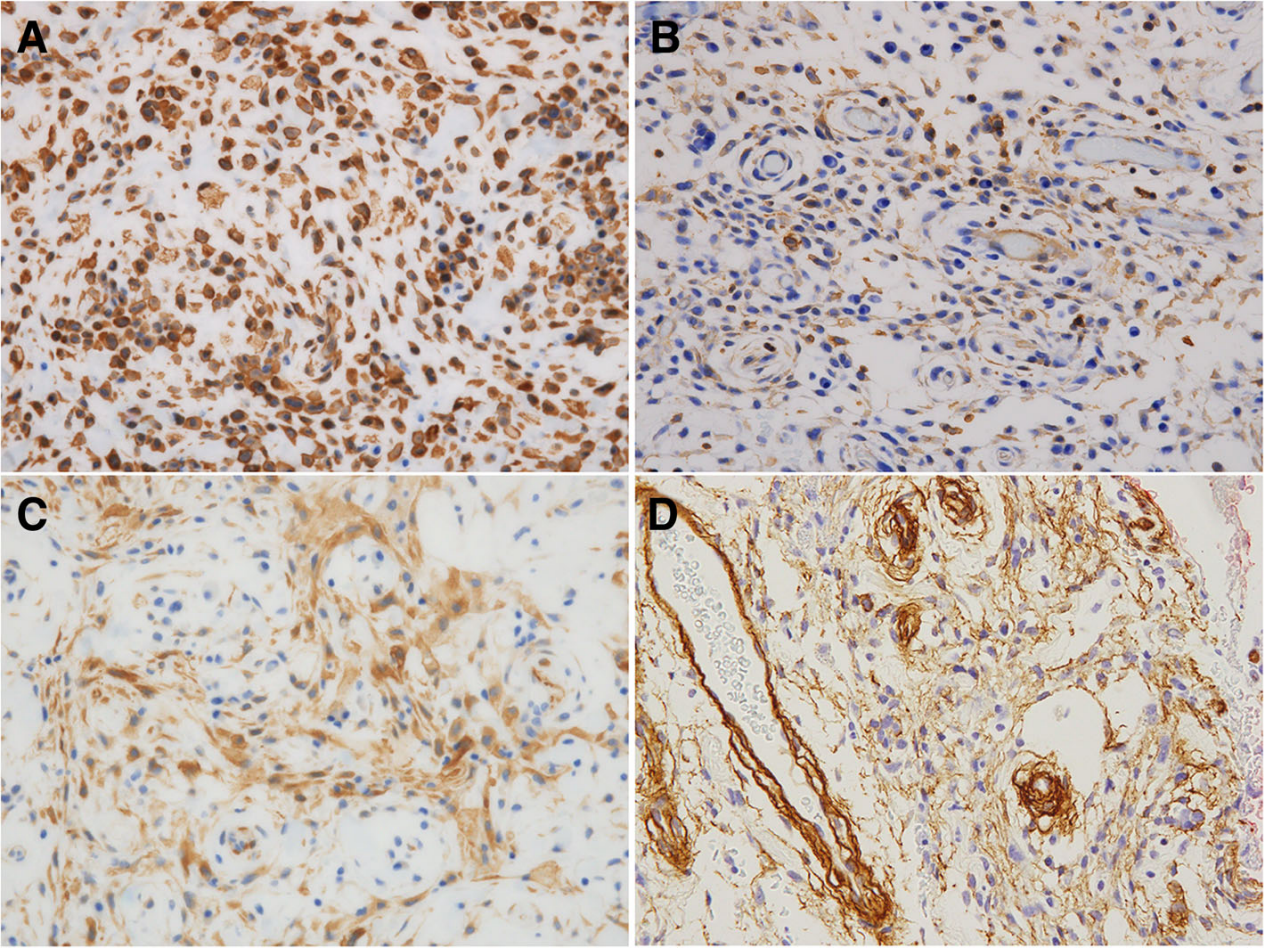
Immunohistochemical findings of the primary glomus tumor. Tumor cells showed positive for vimentin, MSA, Calponin. The area around the tumor cells was positively stained for Type IV collagen in immunohistochemistry. **a** Vimentin, × 400, (**b**) MSA, × 400, (**c**) Calponin, × 400, (**d**) Type IV collagen, × 400

Immunohistochemical staining was positive for vimentin (Fig. 4A), muscle-specific actin/HHF35 (Fig. 4B), and Calponin (Fig. 4C), a protein responsible for binding the actin-binding protein. A hairline-shaped area of positive staining for type IV collagen surrounding the tumor cells was also observed (Fig. 4D). VEGF and D2–40 was also focally positive in tumor cells. In contrast, staining for alpha-SMA, cytokeratin (AE1/AE3), cytokeratin (CAM5.2), CK19, CD34, CD68, p63, S-100, Factor VIII, and desmin, CD56, chromogranin A, synaptophysin was all negative in the tumor cells. Moreover, CD31 was also negative, but we observed many blood cells intervened between tumor nests. The Ki-67 labeling index was almost 1%. No necrosis or tumor invasion into the neurovascular channel was observed. The primary tumor was diagnosed as a glomus tumor based on these findings.

**Fig. 5:**
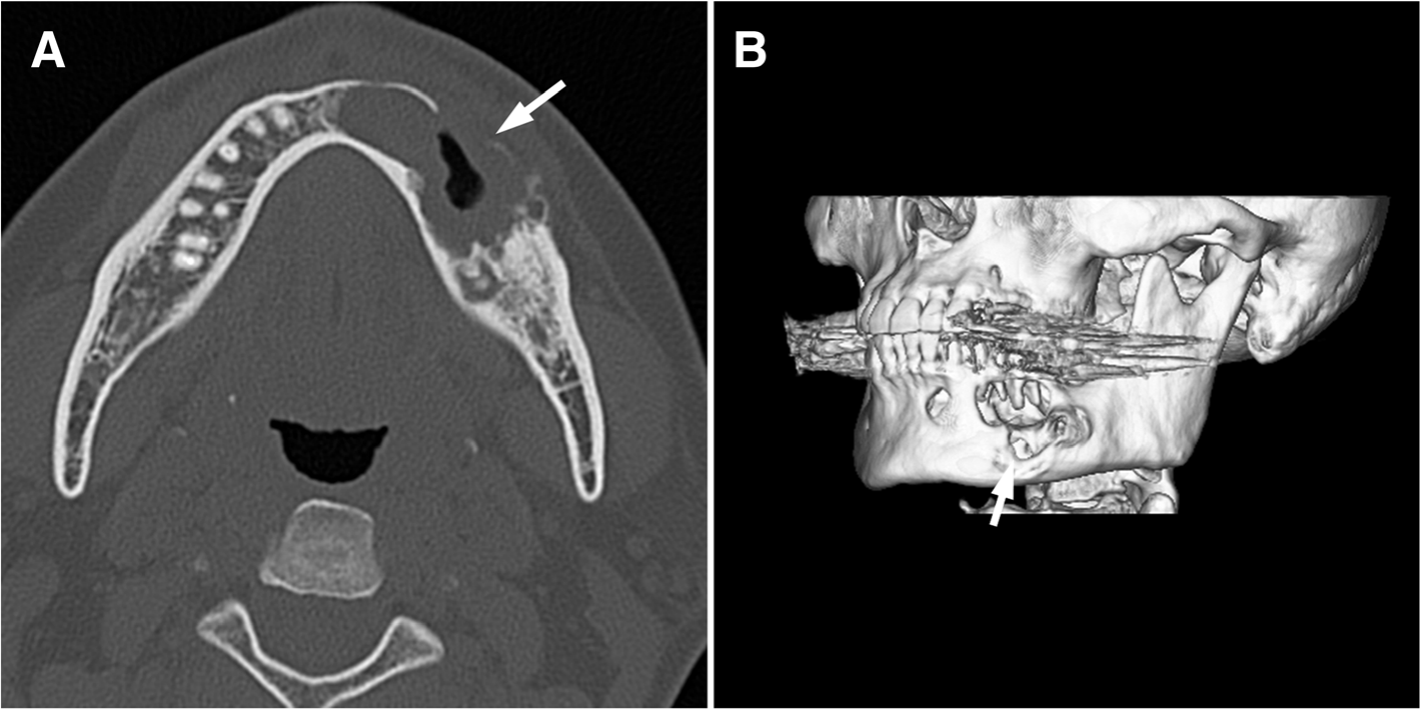
CT images of the recurrent glomus tumor. Axial CT image showing an expanding cortical bone loss (arrow). Three-dimensional volume rendering image. The arrow indicates recurrent location

**Fig. 6:**
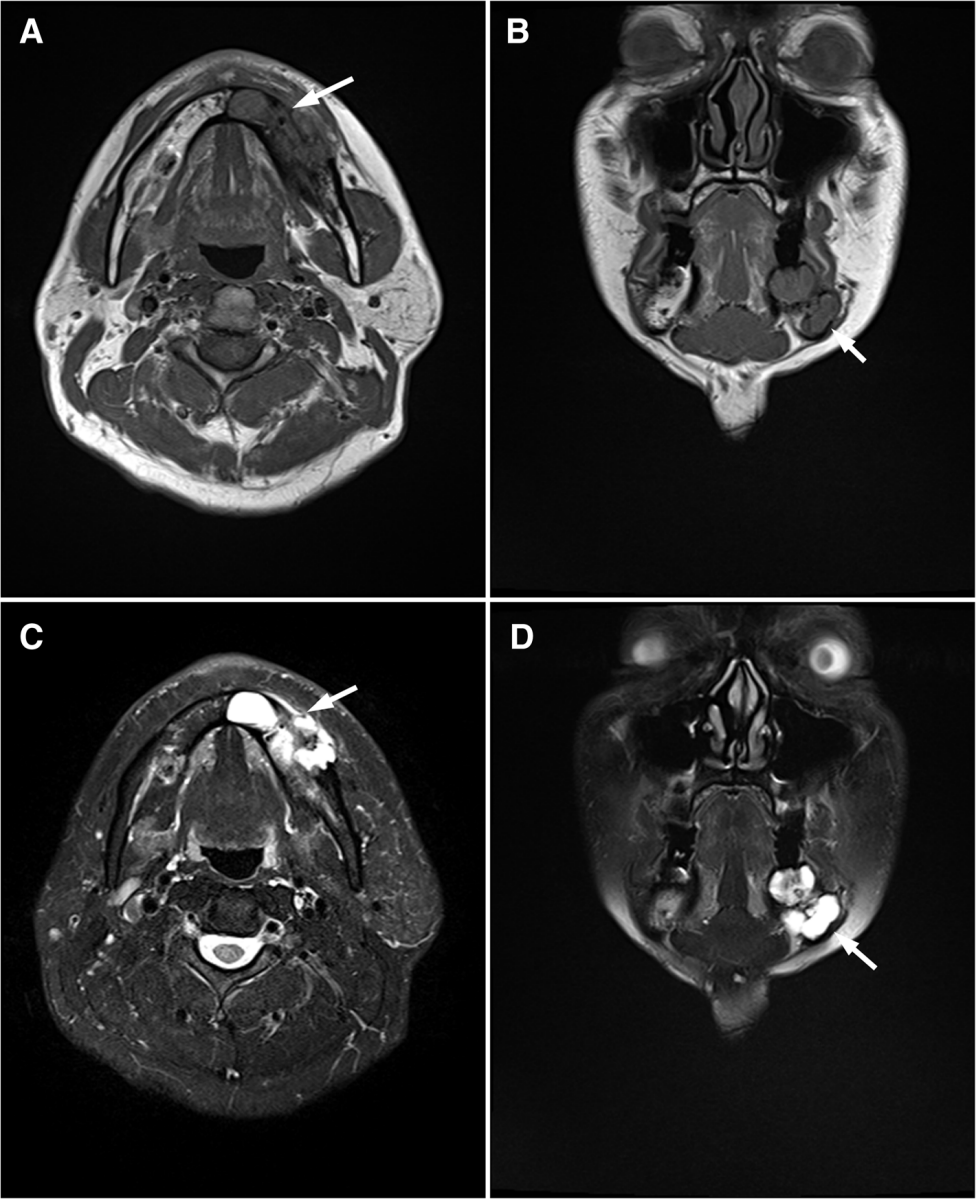
Magnetic resonance images of the recurrent glomus tumor. Axial T1-weighted magnetic resonance image showing the recurrent tumor (arrow). Coronal T1-weighted magnetic resonance image showing the recurrent tumor (arrow). Axial T2-weighted magnetic resonance image showing the recurrent tumor (arrow). Coronal T2-weighted magnetic resonance image showing the recurrent tumor (arrow)

The patient remained symptom-free and manifested no signs of recurrence. However, a recurrent tumor was detected in a panoramic radiograph during an 8-year follow-up. The panoramic radiographs taken earlier, after excision of the primary tumor, showed normal healing process, bone regrowth, and increased radiopacity. The follow-up panoramic radiograph 8 years later depicted the recurrence as a radiolucent expansion in the lower area of the mandible. A CT exam showed an expanding lesion exiting the lower site of the mandible and a thinning buccal cortical bone in contact with the tumor (Fig. 5A, B). In magnetic resonance imaging (MRI), the recurrent lesion showed a lower or compatible signal intensity compared to the muscle in T1-weighted images and a low-to-high inhomogeneous signal intensity in T2-weighted images. The apparent diffusion coefficient in the glomus tumor area was 2.0 × 10^− 3^ mm^2^/sec, suggesting a low cellular density (Fig. 6A-D).

**Fig. 7:**
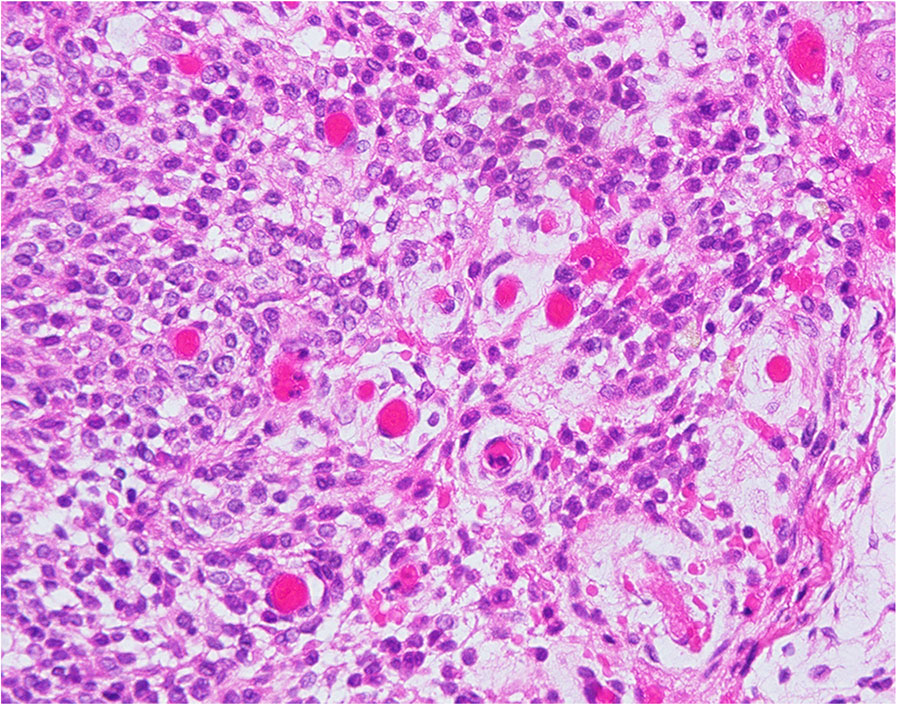
Histologic aspects of the recurrent glomus tumor (H&E stain, × 400). The recurrent tumor had the same microscopic and morphologic features as the primary tumor

The recurrent tumor mass and related teeth were removed under general anesthesia, and the bone surface was shaved. The recurrent tumor had the same microscopic, morphologic, and immunohistochemical features as the primary tumor (Fig. 7). The tumor invaded the medullary cavity of the mandible, involving spongiosa and resorbing compressively the cortex bone around tumor.

The patient has no symptoms of recurrence as of this writing, 2 years after the second operation, and will be followed for the long term to promptly detect any signs of new tumor growth.

The patient is satisfied that the glomus tumor was regulated without resecting the mandibular region. He is pleased that there was no functional deterioration that he was worried about before treatment and there was no aesthetic damage.

Timeline of patient diagnosis and treatment.

1. 4 years before the first visit, the patient had been smashed against his young son’s head.

2. At first visit, the symptom was slight swelling of the left mandible. There was no disturbance of sensitivity. The examinations were performed with X-lay and CT.

3. First surgery was performed and started clinical follow-up after the surgery.

4. 8 years later from first surgery, the recurrence was found by panoramic radiograph. The examinations were performed with CT and MR.

5. Second surgery was performed, and it was passed 2 years with no recurrence from second surgery.

## Discussion

Masson et al. was the first to describe the histologic features of a glomus tumor in an examination of a tumor arising from the glomus apparatus (glomus body) in 1924 [5]. The glomus cell resembles a modified smooth muscle cell of the normal glomus body in the subcutaneous tissue, where it serves as a specialized form of arteriovenous anastomosis for the control of blood pressure and temperature [6, 7]. The vast majority of glomus tumors occur in the distal extremities, particularly the subungual region, hand, wrist, and foot, but rare cases have been reported in almost every location of the body [4].

Only 26 glomus tumors of the oral region and affected bone have been reported in the English-language literature. Our case is rare of reported cases of glomus tumor localized at the mandible in the English-language literature and the case involving the mandible and teeth [7–10]. Previous reports suggest a male predilection for glomus tumors in the oral cavity. Lesions reported within the oral cavity have involved the hard palate, upper lip, gingiva, tongue, buccal mucosa, and mandibular area. Yu et al. reported localized multiple glomus tumors of the oral mucosa and face in the left mandibular area, but none affected the mandible and teeth [11]. The intraosseous location of glomus tumor is also rare [12]. In our case, the CT scan findings showed that the glomus tumor occupied the bone-marrow area and resorbed cortical bone of the mandible. We assume that the glomus tumor was localized in the mandible on the grounds of the CT scan findings, but we could not have the histologic findings that showed that the glomus tumor was formed from the tissue of the intraosseous lesion. Most of the intraosseous glomus tumors reported have been located in the medullary space of a terminal phalanx and encased in cortical bone. The other reported lesions have occurred in the middle phalanxes of fingers, in vertebrae, or in long bones [12].

The glomus tumor in this case was similar to clinical findings with other jaw cysts and benign tumors in mandible. There was no subjective symptom, there was no complain of getting numb at innervated area of inferior alveolar nerve. In the imaging findings, there was no unique findings on the glomus tumor in the mandibular. As a result of the pathological examination, we informed of the glomus tumor.

Glomus tumors are typically treated by surgical excision. While the excision is thought to be curative, the reported incidence of recurrent lesions ranges from 5 to 17% [13, 14]. In a review of recurrent glomus tumors by Gandhi et al., the lesions were often found to be in the same digits as the first excised tumors but at different anatomic locations [15].

It is important that the lesion was resected firmly in treatment of glomus tumor in mandibular. The glomus tumor remaining causes recurrence is what to learn in previous reports [13, 14] and in this case. We assume that an incomplete excision caused recurrence. A complete resection was difficult in our case. Because the patient strongly hoped to preserve his face appearance and the inferior alveolar nerve. We believe that glomus tumor progression can be controlled even if tumor resection is not a mandible area resection in the clinical course of this case.

Morphologically, the tumor cells tend to surround the capillary vessels and contain eosinophilic cytoplasm with smaller round basophilic nuclei [4]. Histologically, the glomus tumor presents in various patterns [12]. The World Health Organization Classification of Tumors has identified three subtypes of glomus tumor respectively based on the predominance of a vascular component (glomangioma), a smooth muscle component (glomangiomyoma), or a cellular component (solid glomus tumor) [4, 12]. In immunohistochemistry, the glomus tumor is positive for smooth muscle actin, pan-muscle actin, and type IV collagen [4].

The tumor cells of both the primary and recurrent lesions of our case appeared around the capillary vessels and presented eosinophilic cytoplasm with smaller round basophilic nuclei. Interestingly, this case showed marked myxoid stromal change in contrast to a typical one. Previous studies reported that some glomus tumors showed extensive myxoid stromal degeneration similar to the present case; moreover, they also suggested that marked myxoid stromal change was correlated with co-expression of actin and CD34 in tumor cells [16, 17]. But in our case, tumor cells showed negative expression for CD34. Pabuccuoglu et al. reported that MMP-9, a member of the matrix metalloproteinase family, produced by glomus tumor cells may be responsible for generating myxoid matrix in stroma [17]. Further analysis will be needed to clarify the mechanism of generating myxoid matrix change in glomus tumor.

In immunohistochemical analysis, both the primary and recurrent lesions were positive for type IV collagen, a characteristic component of the glomus tumor. Almost glomus tumors expressed alpha-smooth muscle actin (SMA) [18]. However, Geraghty et al. reported the case of alpha-SMA-negative glomus tumor [19]. They discussed that the negative staining for the marker represent a variation in the staining pattern [19]. The lesions of this case were negative for alpha-SMA and caldesmon but positive for Calponin and HHF35, which is a muscle-actin-specific antibody and stains the epitope common to alpha-skeletal, alpha-cardiac and gamma-smooth muscle actin, and thus consistent with lesions of myogenic character. Previous studies revealed that glomus tumors showed positivity for MSA (HHF35) in 18 out of 19 cases, Calponin in 4 out of 5 cases, and vimentin in 12 out of 12 cases [18]. Collectively, we consider that the tumor cells have myogenic properties, which don not exclude the possibility that this case is glomus tumor even if staining for alpha-SMA and h-caldesmon are not positive.

In the present study, we found that tumor cells were positive for VEGF and D2–40. Honsawek S. et al. reported that VEGF expression was observed in glomus tumor cells in all examined samples (5 out of 5 cases) [20]. VEGF is a well-known angiogenic factor, and its expression in neoplastic cells may contribute to an abundant vascular network within tumor stroma in glomus tumor. Previous studies reported that staining for D2–40 was negative in glomus tumor cells [21, 22]. The significance of D2–40 expression in glomus tumor cells is unclear. None of the pathological findings on the lesion supported a diagnosis of a vasculature tumor, nervous system tumor, odontogenic tumor, salivary tumor, or any epithelial tumor other than a glomus tumor.

Both the primary and recurrent tumors were finally diagnosed as glomus tumors based on morphologic and immunohistochemical findings. The primary tumor site was judged to be the mandible, in the absence of evidence of metastasis from other tumor sites.

The glomus tumors reported have been mostly benign neoplasms and very rarely malignant. Metastatic disease has been reported in two cases of histologically malignant glomus tumors [23, 24].

In an analysis of 52 cases of atypical and malignant glomus tumors in soft tissue, Folpe et al. reclassified the tumors based on criteria encompassing three new features: deep location, a tumor size larger than 2 cm, and the presence of atypical mitotic figs. [25]. They proposed a classification for glomus tumors with atypical features, subdividing them into four types: malignant glomus tumor, glomus tumor with nuclear atypia only, glomus tumor of uncertain malignant potential, and glomangiomatosis. This classification has since proven useful for the evaluation of atypical and malignant glomus tumors [26]. Atypical and malignant glomus tumors are defined by the following criteria: either 1) a large size (over 20 mm) coupled with deep localization (deep into the muscular fascia), 2) nuclear atypia involving a pleomorphism and prominent nucleoli, or 3) increased mitotic activity (over 5 mitoses / 50 high-power (400 x) fields) and “moderate” to “high” nuclear grade [25]. A “moderate” nuclear grade is characterized by increased nuclear variability and occasional nucleoli, while a “high” nuclear grade is characterized by an enlargement of the nucleus to double or triple the size of a nucleus in an ordinary glomus cell, together with irregular and prominent nucleoli [25]. Malignant glomus tumors have high-level reactivity for MIB-1, p53 [27], and Bcl-2 [28] in immunohistochemical studies.

The classification for malignant glomus tumors proposed by Folpe et al. [25] is based on glomus tumors of the soft tissue and cannot be clearly adapted for tumors of other locations. The criterion “deep into the muscular fascia” is especially difficult to apply to evaluations of tumors not in the soft tissue, given the many possible interpretations of “deep location.” Yet, out of a series of 32 gastrointestinal glomus tumors, all deeply located by definition, only 1 metastasized and 19 were larger than 2 cm [29, 30]. On the other hand, 6 out of 9 deeply located peripheral soft-tissue glomus tumors (all larger than 2 cm) metastasized [25]. The same problem has been discussed for glomus tumors of the sella turcica. Overall, there seems to be a marked difference in the frequency of malignant behavior between deep-seated glomus tumors of the peripheral soft tissue and those located in other “deep” regions [29].

One case reported in the oral region was diagnosed as a malignant glomus tumor of the maxillary gingiva [31] based on lesional cells with atypical tumor cells and heterotypic mitosis regardless of the lesion location. Some have proposed that primary glomus tumors located in bone are unlikely to become metastatic or malignant [12]. One malignant glomus tumor of the bone has been documented, but the details of the case are difficult to ascertain because the report was published in Chinese [32].

Recurrent malignant glomus tumors have been reported, but the rate of recurrence and risk of malignancy cannot be clearly deduced from the literature. The malignant potential of the glomus tumor in the mandibular bone was difficult to evaluate in our case.

Most glomus tumors are solitary and small, with diameters ranging from 10 to 15 mm [8]. Our patient’s tumor measured 45 × 30 × 30 mm and occurred in the mandible bone. This lesion met the malignancy criteria for size, but the tumor had low malignant features of low mitotic activity and absence of significant nuclear atypia. And no recurrence of 8 years was the basis for the lesion being benign. The primary lesion was similar to the recurrent in the morphologic and immunohistochemical findings. And there was no finding suspected of malignancy. The prognosis of this case remains unclear. Close follow-up will be essential, as malignant glomus tumors have been reported on rare occasions.

## Conclusion

The glomus tumor resorbed a bone and teeth roots when the tumor progressed into the mandible. The immunohistochemical features of the tumor were consistent with those described in previous reports. The glomus tumor recurred at 8 years later in this case. The tumor was controlled for 2 years after resecting it again. It is important to completely remove the Glomus tumor.

## References

[1] Giugale JM, Fowler JR (2015). Glomus tumors: a review of preoperative magnetic resonance imaging to detect satellite lesions. Orthopedics.

[2] McDermott EM, Weiss AP (2006). Glomus tumors. J Hand Surg Am..

[3] Looi KP, Teh M, Pho RW (1999). An unusual case of multiple recurrence of a glomangioma. J Hand Surg (Br).

[4] Fletcher DC (2002). Unni KK, Mertens F. Glomus tumors, in World Health Organization classification of tumors. Pathology and genetics of tumors of soft tissue and bone.

[5] Masson P (1924). Le glomus neuromyo-arteriel des regions tactiles et ses tumeurs. Lyon Chirurgical.

[6] Tuncali D, Yilmaz AC, Terzioglu A (2005). Aslan G. multiple occurrences of different histologic types of the glomus tumor*.* J. Hand Surg. A.

[7] Rallis G, Komis C, Mahera H (2004). Glomus tumor: a rare location in the upper lip. Oral Surg Oral Med Oral Pathol Oral Radiol Endod.

[8] Kessaris P, Klimis T, Zanakis S (2001). Glomus tumour of the hard palate: case report and review. Br J Oral Maxillofac Surg.

[9] Ide F, Mishima K, Yamada H, Saito I, Horie N, Shimoyama T, Kusama K (2007). Perivascular myoid tumors of the oral region: a clinicopathologic re-evaluation of 35 cases. J Oral Pathol Med.

[10] Boros AL, Davis JP, Sedgbizadeb PP, Yamashita DD (2010). Glomus tumor: report of a rare case affecting the Oral cavity and review of the literature. J Oral Maxillofac Surg.

[11] Yu HJ, Kwon SJ, Bahn JY, Park JM, Park YW (2000). Localized multiple glomus tumors of the face and oral mucosa. J Dermatol.

[12] Corroller L (2012). Hargunani R, Khashoggi K, Hyes MM, Clarkson PW, Ouellette HA, Munk PL. primary intraosseous glomus tumor in a middle phalanx. Skelet Radiol.

[13] Maxwell G. Patrick, Curtis Raymond M., Wilgis E.F. Shaw (1979). Multiple digital glomus tumors. The Journal of Hand Surgery.

[14] Van Geertruyden J, Lorea P, Goldschmidt D, de Fontaine S, Schuind F, Kinnen L, Ledoux P, Moermans JP. Glomus tumours of the hand: retrospective study of 51 cases. J Hand Surg Br 1996; 21: 257–260. 10.1016/s0266-7681(96)80110-0 8732413

[15] Gandhi J, Yang SS, Hurd J (2010). The anatomic location of digital glomus tumor recurrences. J Hand Surg Am.

[16] Mentzel T, Hügel H, Kutzner H (2002). CD34-positive glomus tumor: clinicopathologic and immunohistochemical analysis of six cases with myxoid stromal changes. J Cutan Pathol.

[17] Pabuççuoğlu U, Lebe B (2008). Matrix metalloproteinase-9 expression in a CD34-positive glomus tumor with myxoid stromal change. Indian J of Dermatol, Venereol and Leprol.

[18] Mravic Marco, LaChaud Gregory, Nguyen Alan, Scott Michelle A., Dry Sarah M., James Aaron W. (2015). Clinical and Histopathological Diagnosis of Glomus Tumor. International Journal of Surgical Pathology.

[19] Geraghty JM, Thomas RW, Robertson JM, Blundell JW (1992). Glomus tumour of the palate: case report and review of the literature. Br J Oral Maxillofac Surg.

[20] Honsawek S, Kitidumrongsook P, Luangjarmekorn P, Pataradool K, Thanakit V, Patradul A (2016). Glomus tumors of the fingers: expression of vascular endothelial growth factor. World J Orthop.

[21] Fukunaga M (2005). Expression of D2-40 in lymphatic endothelium of normal tissues and in vascular tumours. Histopathology.

[22] Kahn HJ, Bailey D, Marks A (2002). Monoclonal antibody D2-40, a new marker of lymphatic endothelium, reacts with Kaposi's sarcoma and a subset of angiosarcomas. Mod Pathol.

[23] Brathwaite CD, Poppiti RJ (1996). Malignant glomus tumor. A case report of widespread metastases in a patient with multiple glomus body hamartomas. Am J Surg Pathol.

[24] Watanabe K, Sugino T, Saito A, Kusakabe T, Suzuki T (1998). Glomangiosarcoma of the hip: report of a highly aggressive tumour with widespread distant metastasis. Br J Dermatol.

[25] Folpe AL, Fanburg-Smith JC, Miettinen M, Weiss SW (2001). Atypical and malignant glomus tumors: analysis of 52 cases, with a proposal for the reclassification of glomus tumors. Am J Surg Pathol.

[26] Nozaki Y, Tomita M, Miyata N, Kumagai K, Kamito K, Kinoshita N, Shindo H (2012). Malignant glomus tumor with repeated recurrence and metastasis: a case report. Orthopedics & Traumatology.

[27] Rodriguez-Justo M, Aramburu-Gonzalez JA, Santonja C (2001). Glomangiosarcoma of abdominal wall. Virchow Arch.

[28] Skelton HG, Smith KJ (1999). Infiltrative glomus tumor arising from a benign glomus tumor: a distinctive immunohistochemical pattern in the infiltrative component. Am J Dermatopathol.

[29] Hanggi D, Adams H, Hans VH, Probst A, Tolnay M (2005). Recurrent glomus tumor of the sellar region with malignant progression. Acta Neuropathol.

[30] Miettinen M (1988). Antibody specific to muscle actins in the diagnosis and classification of soft tissue tumors. Am J Pathol.

[31] Masaki H, Matsuyama A, Kubo T, Nakamura S, Kawagoe T, Hisaoka M, Hashimoto H (2009). A case of maxillary malignant glomus tumor difficult to differentiated from metastatic leiomyosarcoma from the uterus. Jpn J Diagn Pathol.

[32] Sun KK, Xie DH, Song QJ, Shen DH, Qu HY, Liao SL (2007). Malignant glomus tumor of bone: report of a case. Zhonghua Bing Li Xue Za Zhi.

[33] Von Langer R. Gromustumor am harten Gaumen. Wein Med Wochenschr1949; 99:67.

[34] King ES (1954). Glomus tumor. Aust N Z J Surg.

[35] Kirschner H, Strassburg M (1962). Ein am zhanlosen Alveolarfortsatz des Unterkiefers lokalisierter Glomustumor. Dtsch Zahnarzl Z.

[36] Grande R, D'Angelo E (1962). Un tumore glomico del plato duro. Arch Ital Laringol.

[37] Frankel VG (1965). Auftreten eines leimyofibromangioms (Glomangioms) im Wangen-und Jochbogenbereich. Dtsh Zahnartzl Zschr.

[38] Harris R, Griffin CJ (1965). Glomus tumor of the periodontal tissues. Aust Dent J.

[39] Sidhu SS, Subherwal GL (1967). Glomus tumor of palate. Indian Dent Assoc.

[40] Charles NC (1976). Multiple glomus tumors of the face and eyelid. Arch Ophthalmol.

[41] Sato M, Shirasuna K, Sakuda M, Yanagawa T, Yoshida H, Imai J, Maeda N, Kubo K, Yura Y, Miyazaki T, Yagi T (1979). Fine structure of a glomus tumor of the tongue and expression of C type virus in its tumor cells. Int J Oral Surg.

[42] Tajima Y, Weaters DR, Neville BW, Benoit PW, Pedley DM (1981). Glomus tumor (glomangioma) of the tongue: a light and electron microscopic study. Oral Surg Oral Med Oral Pathol..

[43] Saku T, Okabe H, Matsutani K, Sasaki M (1985). Glomus tumor of the cheek: an immunohistochemical demonstration of actin and myosin. Oral Surg Oral Med Oral Pathol.

[44] Ficarra G, Merrell PW, Johnston WH, Hansen LS (1986). Intraoral solitary glomus tumor (glomngioma): case report and literature review. Oral Surg Oral Med Oral Pathol..

[45] Moody GH, Myskow M, Musgrove C (1986). Glomus tumor of lip. A case report and immunohistochemical study. Oral Surg Oral Med Oral Pathol..

[46] Stajcic Z, Bojic P (1987). Intraoral glomus tumour. A case report. J Craniomaxillofac Surg.

[47] Tokiwa S, Sato A, Sakamaki H, Toba H, Kimura Y, Nagumo M, Kohno Y, Tachikawa T (1990). A case of glomus tumor arising in the mandibular gingiva. Jpn J Oral Maxillofac Surg.

[48] Kusama K, Chu L, Kidokoro Y, Kouzu M, Uehara T, Honda M, Ohki H, Sekiwa T, Terakado M, Sato M (1995). Glomus tumor of the upper lip. J Nihon Univ Sch Dent.

[49] Sakashita H, Miyata M, Nagao K (1997). Glomus tumor in the upper lip: a case report. Int J Oral Maxillofac Sur.

[50] Quesada R, Gonzales-Lgunas J, Raspall G (2004). Aggressive glomus tumor of the tongue: report of a case. Med Oral.

[51] Lanza A, Moscariello A, Villani R, Colella G (2005). Glomus tumor of the lower lip. A case report. Minerva Stomatol.

[52] Maeda Y, Irie T, Yamamoto G, Nagoshi Y, Aida T, Takarada M, Matsui Y, Nagumo M, Tachikawa T (2005). Glomus tumor of the palate report of a case and review of the literature. Dental Med Res.

[53] Derand P, Warfvinge G, Tbor A (2010). Glomangioma: A case Presentation. J Oral Maxillofac Surg.

